# Genome Sequences of Neurotropic Lineage III Listeria monocytogenes Isolates UKVDL9 and 2010L-2198

**DOI:** 10.1128/MRA.00228-21

**Published:** 2021-05-06

**Authors:** Taylor M. Albrecht, Zuzana Kucerova, Sarah E. F. D’Orazio

**Affiliations:** a Department of Microbiology, Immunology, and Molecular Genetics, University of Kentucky College of Medicine, Lexington, Kentucky, USA; b National *Listeria* Reference Laboratory, National Center for Emerging and Zoonotic Infectious Diseases, Centers for Disease Control and Prevention, Atlanta, Georgia, USA; University of Maryland School of Medicine

## Abstract

We report the whole-genome sequence of Listeria monocytogenes UKVDL9 and an edited draft genome sequence of L. monocytogenes 2010L-2198. Both are neurotropic lineage III strains; UKVDL9 was isolated from a sheep brain, and 2010L-2198 was isolated from a human subject with rhombencephalitis.

## ANNOUNCEMENT

Listeria monocytogenes is a facultative intracellular bacterium that causes gastroenteritis, sepsis, meningoencephalitis, and rhombencephalitis (1). Although most cases of listeriosis occur in immunocompromised individuals, rhombencephalitis presents in young, otherwise healthy patients. This suggests that some L. monocytogenes strains have virulence factors that promote brainstem infections. We recently reported two L. monocytogenes strains that caused brainstem infections in a mouse model of foodborne listeriosis; both were classified as lineage III by the Institut Pasteur multilocus sequence typing (MLST) database (2). Strain UKVDL9 (sequence type 1140 [ST1140] by MLST analysis), provided by the University of Kentucky Veterinary Diagnostic Laboratory, was isolated from the brain of a sheep with “circling disease.” Strain 2010L-2198 (ST1590, serotype 4c), a human rhombencephalitis isolate, was provided by the CDC. Lineage III strains are not well characterized, and there has been some confusion regarding their origins and pathogenic potential (3, 4). Strain 2010L-2198 is unusual because there are no other isolates in the CDC BioNumerics database (14,788 L. monocytogenes strains collected in the United States, as of 7 January 2021) with the same ST; only three strains (one clinical isolate and two food isolates) have the same ST as UKVDL9.

The whole-genome sequence of strain UKVDL9 was generated *de novo* by ACGT, Inc. (Chicago, IL). A DNA library was prepared from bacteria grown in brain heart infusion (BHI) broth (16 h at 37°C) using the Illumina Nextera XT DNA sample kit and was sequenced on a MiSeq platform (v. 2.6.2.1 software) using a MiSeq 300-cycle microkit (2 × 150 bp), generating a total of 1,926,930 read pairs. An Oxford Nanopore Technologies library was prepared using a ligation sequencing kit (SQKLSK109) and a native barcoding expansion kit (EXP-NBD104) following the manufacturer’s recommended procedure. The median Nanopore read length was 7,630 bases, and the read *N*_50_ value was 17,464 bases. The raw reads were trimmed using Porechop (v. 0.2.3) (5). The genome was assembled into a singular contiguous sequence that was polished using five rounds of Pilon (v. 1.22) (6), and the polished genome was fully circularized using Circlator (v. 1.5.5) (7). Default parameters were used for all software. The 2,885,142-bp genome had a GC content of 38.24% and was annotated by the NCBI Prokaryotic Genome Annotation Pipeline (PGAP) (v. 5.0) (8), which identified 2,722 protein-coding sequences.

A draft genome for strain 2010L-2198 containing 35 contigs was originally reported by the CDC (9). The assembled genome had 23 gaps composed of 4,099 unknown nucleotides. To close some of the gaps, primers were designed to amplify 222- to 1,225-bp fragments by PCR using genomic DNA isolated from bacteria grown in BHI broth (16 h at 37°C) as a template. Amplicons were subjected to Sanger sequencing with both forward and reverse primers (Table 1). This resulted in the addition of 918 nucleotides to the 2,860,258-bp genome (GC content, 38.07%), with most of the remaining gaps being found in 16S and 23S rRNA. Annotation by PGAP v. 5.0 (8) identified 2,739 protein-coding sequences.

**TABLE 1:** Primers used for PCR and sequencing of gaps in the L. monocytogenes 2010L-2198 genome

Gap no.	No. of Ns	Forward primer (5′ to 3′)	Reverse primer (5′ to 3′)	Start position^*a*^	End position^*a*^	Amplicon size (bp)	PCR type^*b*^	Outcome^*c*^
1	16	GCATTAGGTGGAGAGATTGG	CCATAGTATGCTATGCCTATCGTTG	719	734	345	Std.	Consensus, 16 bp added
2	69	GCCTCTAGCTTATATTAGTGTTATGG	CCTTCTCTTTGCTTGTGTTCCG	52435	52503	305	Std.	Consensus, 69 bp added
3	64	CTAGAAATCGCCTGTTTGAGAC	GAGCTGACGGATTACTTGATTC	454894	454957	267	Std.	Consensus, 64 bp added
4	69	CCAGCTTCATTATTCATTTCTCC	CGCCTCCATCTTTTAACCACC	630544	630612	281	Std.	Consensus, 69 bp added
5	149	CCTATGGTTAATTGGTGGTATTC	GTCTCCTGTAAACATTCCACCG	678010	678158	515	Std.	Consensus, 149 bp added
6	18	CCAGAGTTAAGTAATCCAGGATACG	CAAGAGCAGCTGAAAACAACGAC	695832	695849	1,225	Ext.	No PCR product
7	48	GGAAATGCCGTTAATACTGTCAAGC	CTTCCTCAGAATCAGAACCATTTG	946418	946465	490	Std.	Forward sequence only, 48 bp added
8	137	GCGGCACTAACAGTTACAAGTACC	GACTGCAGGAATTGAACCCGCAACC	1055767	1055903	794	Ext.	Consensus, 137 bp added
9	57	GTGTCTTAACCGCTTGACCAAC	GATATTAACACAACAAGGCTTCCCC	1245831	1245983	645	Ext.	No PCR product
10	87	CTAACTGGCGAAGAAGATGAACGCC	GAAAGTGCCTATCCCTAAAGATC	2436980	2437075	490	Std.	No DNA sequence
11	9	GGTTTGTATATCCTGAGCATGGAC	CTATTCCCAACTATGAAACACCGCG	2181852	2181860	738	Ext.	No PCR product
12	13	CTTGCGCCATCGTGTACTAAGAC	GGGATTGCTTCTACGCCATCTTC	2724201	2724213	897	Ext.	No PCR product
13	43	CAAAGTAATCGCAAAATATTGGGCATCTGG	CCCTTCAACTCAATATTGTCTCGC	2781753	2781795	425	Ext.	Consensus, 43 bp added
14	117	CCCTCCTCAATTCAACTCCATCTTTTG	GGGGATAGAAACTAAGCCAGATGATTGG	2803645	2803911	414	Std.	Forward sequence only, 117 bp added
15	33	GGTAATAGAGCTGAGATCGGAAAGC	CTTGTCCAACTCAATTTGCCC	2806090	2806122	222	Std.	Consensus, 33 bp added
16	63	GAGGTATCATGATAGCGCAATTG	CCAAAGACATTTCTGACTGCGAG	2807070	2807132	398	Std.	Reverse sequence only, 63 bp added
17	1	GATAGCACTGCACCCGTCGC	CACCAAGTTCGGTCGCATTTCAC	1535537	1535537	617	Ext.	No PCR product
18	1	CCAACTGAGCTAAAGCGGCAGC	GGCGAAAAAGTTGCTGCAGACTTAG	1769200	1769200	642	Ext.	No PCR product
19	62	GCACTGTCAGGAAATACACTATCATAGGTGTCCCC	CGCTATCACCTGAAACCGAGGCAC	2079565	2079626	1,011	Ext.	Forward sequence only, 62 bp added
20	48	CATCTAATTCTTGACTGGGAGCAACC	CATGACGAATGCGCGACCAGATG	2327984	2328318	1,109	Ext.	Consensus, 48 bp added
21	17	CGCAGCACATATGACCGGTTATGAG	CCTGTTTGTTAGTGCAAGCGTAGGG	2639718	2640084	912	Ext.	No PCR product
22	1	GGTACGAATTGAAGCCCCAGTAAACGG	GGCTTTCTGGTTAGATACCGTCAAGG	2852978	2852978	343	Ext.	No PCR product
23	93	GCAAGGCACCATGCTTGAAGC	CCCAATCTCTCCACCTAATGCTCAGTG	1	93	915	Ext.	No DNA sequence

a Nucleotide positions refer to the original fasta sequence deposited as GenBank accession no. AAKGBO000000000.1. The newly edited version submitted to GenBank under accession no. CP069380 is the reverse complement of that sequence and was rearranged to start with the *dnaA* gene by convention.

b PCR conditions used were as follows: standard (Std.), 2 min at 94°C; 30 s at 94°C, 30 s at 57 to 61°C, and 45 s at 72°C for 30 cycles; and 2 min at 72°C; extended (Ext.), 2 min at 94°C; 45 s at 94°C, 45 s at 61°C, and 1 min at 72°C for 35 cycles; and 2 min at 72°C. For all reactions, Invitrogen Platinum II Hot-Start PCR master mix (2×) containing Platinum II *Taq* Hot-Start DNA polymerase was used.

c Consensus, DNA sequences for the PCR product were obtained from both the forward and reverse primers; no DNA sequence, a PCR product of the expected size was obtained, but it generated noisy reads.

### Data availability.

Raw reads for UKVDL9 were deposited in the Sequence Read Archive (SRA) as accession no. SRX10056804 (Illumina) and SRX10056805 (Nanopore); the circular genome sequence can be found as GenBank accession no. CP065028. Raw reads for 2010L-2198 were deposited as BioProject no. PRJNA212117; the revised draft genome is GenBank accession no. CP069380.1.
